# Continuing or adding IL-2 in patients treated with antiretroviral therapy (ACTG Protocol A5051, a rollover trial of ACTG Protocol A328)

**DOI:** 10.1186/1742-6405-7-30

**Published:** 2010-08-05

**Authors:** Ronald J Bosch, Richard B Pollard, Alan Landay, Evgenia Aga, Lawrence Fox, Ronald Mitsuyasu

**Affiliations:** 1 Harvard School of Public Health, Boston MA, USA; 2 University of California, Davis Medical Center, Sacramento, CA, USA; 3 Rush University Medical Center, Chicago IL, USA; 4 National Institute of Allergy and Infectious Diseases, Bethesda, MD, USA; 5 University of California, Los Angeles, Los Angeles, CA, USA

## Abstract

**Background:**

Effective antiretroviral therapy reduces HIV-1 RNA levels, improves CD4 T-cell counts, and lowers the risk of opportunistic infections and malignancies. Interleukin-2 (IL-2) has been shown to increase CD4 T-cell numbers mainly by expanding CD4 cells and by prolonging their half-lives. HIV-infected patients previously enrolled into A328 had been randomized to antiretroviral therapy (ART) alone or ART followed by IL-2. In A5051, 53 patients from A328 who had previously received IL-2 were allowed to continue IL-2 for an additional 80 weeks; 27 patients who had received ART alone received IL-2 for 80 weeks.

**Results:**

The patients previously receiving IL-2 continued to have elevated CD4 levels with extended use of IL-2. The prior ART-alone recipients had increases in CD4 levels to comparable levels as the prior IL-2 recipients (median 804 versus 847 cells/mm^3 ^at week 72; 60% versus 9% had >50% increase in A5051 to week 72, p < 0.001). Those who had previously received IL-2 required fewer IL-2 cycles to maintain their CD4 T-cell counts compared to those newly initiating IL-2. The treatments were well tolerated with no significant differences in toxicity or discontinuations between those newly versus previously receiving IL-2. There were few clinical events observed.

**Conclusions:**

Although sustained CD4 T-cell count increases were seen with IL-2 administration as in other studies, the absence of clinical benefit in two recent randomized trials has demonstrated no apparent role for IL-2 as a therapy in HIV disease.

**Trial Registration:**

A5051 ClinicalTrials.gov Identifier: NCT00000923.

## Background

In HIV infection, CD4 cell number progressively decreases, predisposing affected individuals to the development of opportunistic infections or malignancies if they are left untreated. Effective antiretroviral therapy reduces HIV-1 RNA levels, improves CD4 counts, and lowers the risk of opportunistic infections and malignancies. Interleukin-2 (IL-2) has been shown to increase CD4 T-cell numbers mainly by expanding CD4 cells and by prolonging their half-lives [1-3].

AIDS Clinical Trial Group (ACTG) study A5051 was an open-label study that explored continuation therapy with IL-2 for patients who had participated in A328 [4]. A328 enrolled patients with little or no prior antiretroviral experience and CD4 T cell counts between 50 and 350 cells/mm^3 ^who were then treated with a 12-week course of protease inhibitor-containing antiretroviral therapy. If their HIV-1 RNA was less than 5,000 copies/ml, they were randomized to continue antiretroviral therapy (ART) alone or with either IV or subcutaneous IL-2. The study showed that IL-2 significantly expanded CD4 cell numbers, did not increase HIV-1 RNA levels and appeared to reduce the number of AIDS-defining events [4].

The current study (A5051) was designed to investigate the effects of continuing subcutaneous IL-2 in patients receiving IL-2 in A328 and to determine the activity of IL-2 in patients who had previously been treated with antiretroviral therapy alone for 84 weeks.

## Methods

Patients were eligible for A5051 if they had completed ≥84 weeks of A328 [4]. Those with prior IL-2 must have had a CD4 increase at week 60 or 72 of at least 25% of their A328 week 12 CD4 level (timepoint of IL-2 initiation), and all patients had a plasma HIV RNA ≤5,000 copies/ml before the IL-2 phase of A5051. Patients who received IV IL-2 in A328 were allowed to switch to subcutaneous IL-2 during that study. Most patients previously treated with IL-2 had received subcutaneous IL-2 prior to enrolling in A5051 [4].

Patients with >5,000 copies/ml of HIV-1 RNA or those without prior IL-2 who had HIV-1 RNA ≤5,000 copies/ml could change their antiretroviral regimen and then begin IL-2 after documenting HIV RNA ≤5,000 copies/ml. Subjects were then followed for 80 weeks. All subjects provided written informed consent and the study was approved by the institutional review boards of each participating site.

The primary objective was to determine the long-term safety and efficacy of IL-2 in maintaining or increasing CD4 T-cell counts in patients who had previously been in A328. Other objectives were to describe the effect of IL-2 on virologic breakthrough, to examine the durability of immunologic changes on long term IL-2, and to determine if there were differences in those that started on IL-2 after 84 weeks of antiretrovirals alone as compared to those begun after 12 weeks of ART.

CD4 and CD8 T-cell counts were measured every 8 weeks during the 80-week trial. HIV-1 RNA testing was performed at an ACTG certified laboratory every 24 weeks. Routine hematology and chemistries were performed prior to and at day 5 and 30 of each IL-2 cycle. Pregnancy tests were performed on female patients 2 weeks prior to entry and 2 weeks before each cycle. A skin test battery consisting of PPD (Connaught), tetanus toxoid (Connaught) mumps (Connaught) and candida albicans (ALK laboratories) was performed every 24 weeks.

An immunology substudy measured advanced flow markers and lymphoproliferation responses at cycle 2, 4, 7 and at the time of the final evaluation using ACTG consensus methods that used fluorochrome-labeled monoclonal antibodies to CD3, CD4, CD8, CD45RA, CD62L, CD38 and HLA-DR. Proliferation responses were measured to phytohemaglutenin (PHA), tetanus toxoid, candida and HIV antigens using ACTG consensus methods.

IL-2 was administered at 4.5 million units SC BID for 5 days every 8 weeks for the first three cycles for patients without prior IL-2. Subsequent cycles could be extended by 8-week increments for a maximum of 24 weeks as long as CD4 counts were >500 cells/mm^3^. The dose could be reduced to 2.5 million units twice daily at the subsequent cycle if IL-2 was interrupted due to toxicity or intolerability. Patients with prior IL-2 were administered IL-2 at 4.5 million units SC BID (or 2.5 million units BID if reduced to this level in A328) every 8 weeks. Starting with the first cycle, longer intervals were allowed if the CD4 T-cell count stayed above 500 cells/mm^3^.

The primary efficacy endpoint of A5051 was a CD4 cell count after 48 or 72 weeks that was 50% above the CD4 cell count at A5051 baseline, comparing those newly receiving IL-2 versus prior IL-2 recipients. Fisher's exact test compared proportions. For ordinal and continuous variables, the Wilcoxon rank-sum test was used. The log-rank test compared time-to-event data.

## Results

Eighty-one patients enrolled into A5051; 80 directly into the IL-2 phase and one after a change in antiretroviral regimen. One patient admitted to not taking antiretrovirals during A328 and was excluded from efficacy analyses, but was included for the toxicity assessment. Patients enrolled from 19 sites between April 1999 and November 2000; 28 patients enrolled in the immunology substudy. For the efficacy analysis, 27 of 52 (52%) patients in the ART-only arm of A328 enrolled. Comparable percentages of those with prior IL-2 enrolled, with 28 of 53 (53%) patients randomized in A328 to intravenous IL-2 enrolling and 25 of 54 (46%) of patients randomized in A328 to subcutaneous IL-2.

The median age was 40 (25^th ^- 75^th ^percentiles: 35-48). There were only 4 women; 47 were white non-Hispanic, 16 were black non-Hispanic, 14 were Hispanic and 3 were Asian, Pacific Islander or American Indian. The median baseline CD4 T cell counts were 832 (595-1320) and 466 (367-592) cells/mm^3 ^for patients with and without prior IL-2, respectively. Previously IL-2-treated subjects had greater increases from A328 week 12 to A5051 baseline in total lymphocyte counts (p < 0.001), CD4 T cell counts (p < 0.001), CD8 T cell counts (p < 0.017) and CD4 T cell percentage (p < 0.001).

The majority of patients 65/80 (81%) completed the study; six were lost to follow-up, one died (cause of death unknown), and three without prior IL-2 discontinued the study due to IL-2-related side effects. The majority of patients (83%) were on stavudine, lamivudine and indinavir (n = 47) or zidovudine, lamivudine and indinavir (n = 19); the other regimens were stavudine/didanosine/indinavir (n = 5), zidovudine/didanosine/indinavir (n = 1), stavudine/didanosine/indinavir/nevirapine (n = 1), stavudine/didanosine/nelfinavir (n = 3), stavudine/lamivudine/nelfinavir (n = 2), abacavir/efavirenz/nelfinavir (n = 1) and zidovudine/lamivudine/nelfinavir/nevirapine (n = 1). Most patients (64/80, 80%) completed study treatment (81% and 78%, for those with and without prior IL-2) and no significant difference was seen between the groups in the time to treatment discontinuation (p = 0.96).

With regard to IL-2 dosing, 79 patients received at least one cycle of therapy; one patient with previous IL-2 never met the criteria for starting IL-2 (CD4 count ≤500 cells/mm^3^). The median total dose was 45 million international units for all cycles. Previously IL-2-treated patients had longer times between cycles because they did not meet the CD4 criterion for a subsequent cycle, resulting in a median (25^th^-75^th ^percentiles) of 5 (3-7) cycles for patients without prior IL-2, and 4 (3-5) cycles for patients with prior IL-2. In patients without prior IL-2, 16/27 (59%) received ≥5 cycles versus 18/53 (34%) patients with prior IL-2.

More occurrences of grade ≥3 signs/symptoms and grade ≥3 or higher laboratory abnormalities were seen in those newly receiving IL-2, but differences were not statistically significant (p = 0.13 and p = 0.072). There was only one AIDS-defining event which was Kaposi's sarcoma diagnosed at week 32 and only one HIV-related event, oral hairy leukoplakia at week 42, both in patients with prior IL-2.

**Figure 1:**
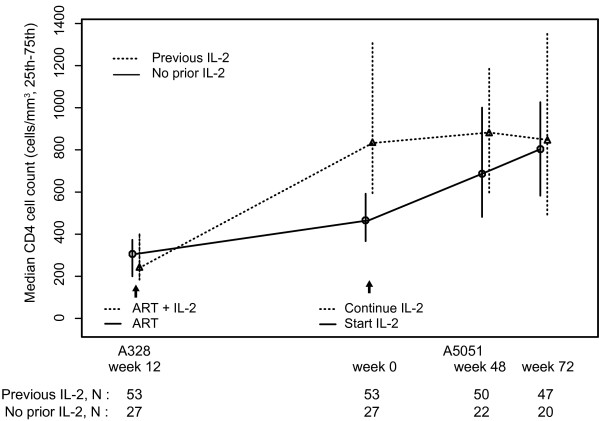
**Median CD4 T cell counts over time for the participants in A5051**. The 'Previous IL-2' group initiated IL-2 at week 12 of A328. The 'No prior IL-2' group initiated IL-2 in A5051. Vertical bars represent 25^th ^and 75^th ^percentiles.

There were significant differences in the primary endpoint. In those newly receiving IL-2, 12/22 (55%) had 50% increases in CD4 count at week 48 and 12/20 (60%) at week 72, compared to 2/50 (4%, p < 0.001) and 4/47 (9%, p < 0.001) in those with prior IL-2. Prior IL-2 recipients started with higher CD4 counts and maintained these levels, in comparison to those newly receiving IL-2 whose CD4 counts increased and attained comparable levels at week 72 (median 847 versus 804 cells/mm^3^, 74% versus 90% ≥ 500 cells/mm^3^; Figure 1).

There was no evidence of increased virologic breakthrough, defined as two consecutive HIV-1 RNA levels ≥5000 copies/ml. Three subjects, all previously IL-2-treated, met this criterion; two had pre-study HIV-1 RNA levels between 3000 and 4500 copies/ml and one had <50 copies/ml. Among 64 subjects who entered with HIV-1 RNA <75 copies/ml, four subsequently had two consecutive measurements ≥ 1000 copies/ml (two subjects with and two without prior IL-2).

Skin test reactivity was examined by the number of positive responses (induration ≥10 mm). At week 48, more positive responses were seen in those newly receiving IL-2 (4/8 versus 1/19 with ≥2 positive responses, p = 0.046, analyzing those with data for all four antigens) although this was not confirmed at week 72 (2/8 versus 2/16, p = 0.96).

In the immunology substudy, there were no significant differences in LPA responses to the tested antigens between patients newly versus previously receiving IL-2 (p > 0.5 at both week 48 and 72).

**Figure 2:**
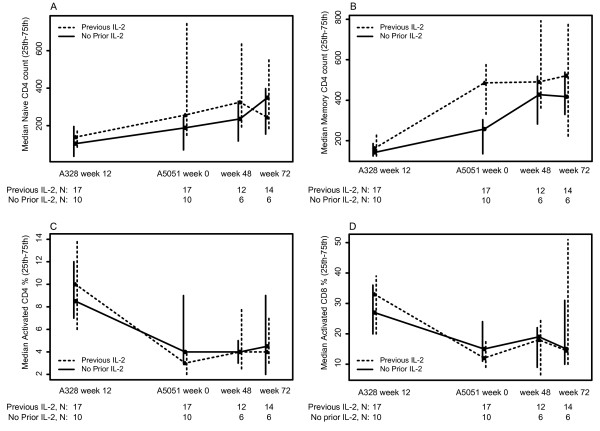
**Median CD4 and CD8 T cell subsets over time for the participants in A5051**. (A) Naïve (CD45RA+/CD62L+) CD4 T-cell counts, (B) Memory (non-naïve: CD45RA- or CD62L-) CD4 T cell counts, (C) Percentage activated (CD38+/HLA-DR+) CD4 T-cells and (D) Percentage activated (CD38+/HLA-DR+) CD8 T-cells.

Despite small sample sizes, longitudinal assessment of CD4 and CD8 subsets showed that the number of naive (CD45RA+/CD62L+) CD4 cells rose gradually in both groups during A328 and A5051 (Figure 2). Memory (non-naïve) CD4 counts increased in response to IL-2 in A328 as compared to a more modest increase in the antiretroviral-alone arm, which was followed by an increase after IL-2 in A5051. The prior IL-2 recipients maintained CD4 memory cells during A5051. There were significant decreases in CD4 and CD8 activation markers (% CD38+/HLA-DR+) during A328 in both the IL-2 and antiretroviral-alone groups but little change during A5051.

## Discussion

As a rollover trial to A328 [4], A5051 produced additional data on the long-term safety and efficacy of IL-2. With regard to safety, the dosages utilized, which averaged 4.5 million international units BID for 5-day cycles, were relatively well tolerated. There was some increase in both symptoms and laboratory abnormalities that appeared to be higher in those receiving IL-2 for the first time but these differences did not reach statistical significance.

The side effect profile was actually better than observed in A328 wherein the starting doses were higher in both the IV groups and the subcutaneous group. However, the majority of patients in that trial had been reduced to the dosage utilized in A5051 and had been switched to subcutaneous IL-2. Patients who had received IL-2 in A328 tolerated its continuation for the additional period in A5051, although A5051 may have selected for individuals better able to tolerate IL-2. In those newly initiating IL-2, more than half received ≥5 cycles. Overall, the data suggest that this dosage is relatively well tolerated.

With regard to the primary endpoint, those newly receiving IL-2 had a significantly greater likelihood of a 50% increase in CD4 T-cell counts at 48 and 72 weeks. Previously IL-2-treated patients had continued, relatively constant, higher CD4 T-cell counts which had been previously increased during A328. This may be due to a form of homeostatic effect which keeps CD4 counts induced by IL-2 within a finite range, preventing CD4 counts from going into a supernormal range. Patients who had received IL-2 in A328 also had fewer cycles of IL-2 than those newly initiating IL-2 in this study.

There was no evidence that administration of IL-2 increased the rate of virologic breakthrough as measured by increases in HIV-1 RNA levels, consistent with other studies showing viral suppression in 80-90% of patients treated with IL-2 and ART, similar to those receiving ART alone [4,5].

The advanced flow data suggests IL-2 has a greater influence on CD4 memory T-cells in our study population than on naive T-cells as has been reported [6]. The influence of IL-2 is two-fold: memory cells are increased in number and their longevity is prolonged [2,3]. The lesser increase in naïve versus memory CD4 T-cells in those newly receiving IL-2 in the present study may be due to subjects' prior immune reconstitution on ART and their higher pre-IL-2 total CD4 T-cell counts (median 466 cells/mm^3^) and naïve CD4 T-cell counts as compared to earlier investigations (mean pre-IL-2 CD4 T-cell count: 300 cells/mm^3^)[6]. Decreases were seen in both CD4 T-cell and CD8 T-cell activation markers, but patterns were similar for those first receiving IL-2 in A328 versus A5051.

Although some evidence was seen that new receipt of IL-2 enhanced skin test reactivity, the recent findings of the ESPRIT and SILCAAT randomized trials showed no clinical benefit from long-term IL-2 when added to antiretroviral therapy [5]. This is in contrast to A328 [4], which showed an apparent decrease in AIDS-defining and HIV-related infections in the IL-2 treated group.

## Conclusions

This study showed that as compared to a group of patients that had tolerated and responded to treatment with concurrent antiretroviral therapy and IL-2 in a previous study, patients newly exposed to IL-2 after initial ART achieved similar CD4 T-cell numbers after 72 weeks. Median CD4 levels above 800 cells/mm^3 ^were maintained in the previously IL-2 treated patients, with fewer cycles than in those newly receiving IL-2. But considering the two large randomized trials and their findings that IL-2 therapy in HIV-infected patients receiving antiretroviral therapy showed no clinical benefit but higher rates of serious adverse events [5], these studies do not support a role for IL-2 in HIV infection.

## Competing interests

RBP was a consultant for BMS (Bristol-Myers Squibb) during the study and is now on their speakers bureau. The other authors declare no competing interests.

## Authors' contributions

RJB and EA performed the statistical analyses. RBP, AL, LF and RM conceived of the study, and participated in its design and implementation. All authors contributed to and approved the final manuscript.
